# Colorectal adenosquamous carcinoma: genomic profiling of a rare histotype of colorectal cancer

**DOI:** 10.1007/s00428-023-03517-6

**Published:** 2023-02-15

**Authors:** Valentina Angerilli, Paola Parente, Gianluca Businello, Alessandro Vanoli, Michele Paudice, Giovanni Perrone, Giada Munari, Ilaria Govoni, Giuseppe Neri, Elena Rebellato, Paola Parrella, Federica Grillo, Luca Mastracci, Matteo Fassan

**Affiliations:** 1 Department of Medicine (DIMED), Surgical Pathology Unit, University Hospital of Padua, Padua, PD Italy; 2 Pathology Unit, Fondazione IRCCS Casa Sollievo Della Sofferenza, San Giovanni Rotondo, FG Italy; 3 Department of Molecular Medicine, Anatomic Pathology Unit, University of Pavia, Viale Camillo Golgi, 19, 27100 Pavia, PV Italy; 4 Fondazione IRCCS San Matteo Hospital, Pavia, PV Italy; 5 Ospedale Policlinico San Martino IRCCS, Genoa, GE Italy; 6 Department of Surgical Sciences and Integrated Diagnostics (DISC), Anatomic Pathology, University of Genova, Genoa, GE Italy; 7 Veneto Institute of Oncology IOV - IRCCS, Padua, PD Italy; 8 Laboratory of Oncology, Fondazione IRCCS Casa Sollievo Della Sofferenza, San Giovanni Rotondo, FG Italy

**Keywords:** Next-generation sequencing, Colorectal carcinoma, Adenosquamous carcinoma

## Abstract

Colorectal adenosquamous carcinoma (ASC) is exceedingly rare, comprising less than 0.1% of all colorectal malignancies, and is characterized by an aggressive disease course, with a higher metastatic rate and worse outcome than conventional colorectal adenocarcinoma. A comprehensive molecular profile of this group of neoplasms is still lacking. A total of 22 cases of colorectal ASCs (with 22 primary lesions and 7 metastases matched with 4 primaries) were subject to NGS targeting 67 cancer-related genes (VariantPlex solid tumor; Archer). Mismatch repair (MMR), p53, and ^V600E^*BRAF* status were also investigated by immunohistochemistry. In 28 of 29 (96.6%) ASC samples, at least one single-nucleotide variant (SNV) or copy number variation (CNV) was detected. Among the 22 primary tumors, the most frequently mutated genes were *TP53* (59.1%), *APC* (40.9%), *KRAS* (27.3%), *BRAF* (13.6%), and *GNAS* (9.1%). Only 1/22 (4.5%) primary ASC was MMR-deficient (MMRd) and harbored a *BRAF* mutation. Limited differences in SNVs were observed between primary and metastatic diseases. This study sheds light on the molecular landscape of colorectal ASCs. According to our data, the genomic profile of colorectal ASC is similar to that of conventional colorectal carcinoma, with significant druggable genetic alterations. Further studies are required to understand the more aggressive clinical behavior of this neoplasm.

## Introduction

Adenosquamous carcinoma (ASC) of the colon-rectum is a rare neoplasm, representing approximately 0.5–1% of colorectal malignancies [1]. ASC has been described in patients with ulcerative colitis and has been associated with paraneoplastic hypercalcemia. No significant difference in sex, age, and ethnicity between ASC and conventional colo-rectal cancer (CRC) has been reported [2].

From the histopathological point of view, ASC is a malignant neoplasm composed of separate and coexisting elements of squamous cell carcinoma and adenocarcinoma with various grades of differentiation. According to the 2019 edition of the Word Health Classification (WHO) of Gastrointestinal Tumors, colorectal ASC to be classified as such, similarly to esophageal ASC, should contain at least 20% of each component [1].

Colorectal ASC is characterized by an aggressive disease course, with a higher metastatic rate and worse outcome compared to conventional CRC, and advanced tumor stage at onset. A recent study by our group demonstrated that the poor prognosis of this histotype is associated with aggressive pathologic features, and the two components show different immunohistochemical profile, with nuclear staining of cyclin D1 predominately in the squamous component and nuclear staining of β-catenin in the glandular component, respectively [3].

Due to the rarity of this histotype, consistent molecular data are lacking. A previous study has investigated the molecular profile of a case of colorectal ASC arising in the context of Lynch syndrome by microdissecating the glandular and squamous components and found a similar mutational profile, with mutations in *KRAS*, *PIK3CA*, *GNAS*, and *TP53* [4]. Another study reported *KRAS* and *CTNN1B* mutations both on primary and metastatic ASCs [5].

The aim of this study is to provide a comprehensive molecular evaluation of a multi-institutional series of primary and metastatic colorectal ASCs.

## Materials and methods

### Case selection

We retrospectively collected 22 cases of colorectal ASC and 7 matched metastases obtained from 4 cases. Cases were collected from the surgical pathology units of Padua University Hospital (Padua, Italy), Ospedale Policlinico San Martino IRCCS (Genoa, Italy), Fondazione IRCCS Casa Sollievo della Sofferenza (San Giovanni Rotondo, Italy), and Fondazione IRCCS Policlinico San Matteo (Pavia, Italy) between 1992 and 2022.

Hematoxylin and Eosin-stained slides were jointly reviewed by three expert gastrointestinal pathologists (M.F., L.M., and F.G.). Only ASC cases showing at least 20% of both components, as specified in the WHO classification 2019 [1], were enrolled in the study. All information regarding human tissue was managed using anonymous numerical codes, and all samples were handled in compliance with the Declaration of Helsinki (https://www.wma.net/what-we-do/medical-ethics/declaration-of-helsinki/).

### Immunohistochemistry (IHC)

IHC was performed using the bond polymer refine detection kit (Leica Biosystems, Newcastle upon Tyne, UK) in the BOND-MAX system (Leica Biosystems). Four-micrometer-thick FFPE sections were incubated with the following primary antibodies: MLH1 (clone ES05; Dako), PMS2 (clone EP51; Dako), MSH2 (clone FE11; Dako), MSH6 (clone EP49; Dako), ^V600E^*BRAF* (clone VE1; Ventana), and p53 (clone DO-7; Dako).

p53 and ^V600E^*BRAF* immunostaining were performed only if a *TP53* or *BRAF* mutation was found by mutational analysis.

Nuclear immunostaining for MLH1, PMS2, MSH2, and MSH6 was evaluated following the GIPAD-SIAPeC criteria [6] to identify mismatch repair deficiency (MMRd) and mismatch repair proficiency (MMRp) profile.

p53 was considered as aberrant in the presence of complete loss or diffuse and strong nuclear immunostaining in neoplastic cells.

^V600E^*BRAF* was considered positive in the presence of cytoplasmic positivity.

### Targeted next-generation sequencing (NGS) by Archer® VariantPlex® solid tumor

Two experienced pathologists (M.F. and P.P.) carefully marked a representative area for every tumor sample, to ensure that each area contained > 50% of neoplastic cells. Microdissection of the two components was not possible as the two histotypes were substantially intermingled in most of the cases. Five consecutive 10-μm-thick sections from each FFPE sample were obtained. The previously marked areas were manually (i.e., scalpel blade-assisted) microdissected from adjacent tissue.

The QIAmp FFPE tissue Kit (Qiagen) was used to isolate DNA from the dissected material, according to the manufacturer’s instructions. The concentration and the purity of DNA sample were evaluated by Qubit® 3.0 fluorometer and the Qubit® DNA BR assay kit (Thermo Fisher Scientific).

The Archer® VariantPlex® solid tumor panel is based on a targeted enrichment method called anchored multiplex PCR (AMP). The panel allows the detection of single-nucleotide variant (SNV) of 63 target genes, and the analysis of copy number variation (CNV) for 44 genes, frequently associated with cancer.

Only samples with good DNA quality, assessed using the Archer PreSeq DNA QC assay, have been used to create the libraries, according to the manufacturer’s instructions (ArcherDX). For each patient, 50–200 ng of total DNA were fragmented and amplified using specific primers provided by the manufacturer. Libraries were quantified using the KAPA library quantification kit (Roche) and pooled to equimolar concentration. Next-generation sequencing (NGS) was performed on a NextSeq-550 Platform (Illumina), and results were analyzed using the Archer® Analysis v6.0 software.

SNVs were considered pathogenic based on previous interpretations of exonuclease domains (ClinVar). However, for variants of uncertain significance, VarSome was used to determine potential pathogenicity [7]. Only pathogenetic and likely pathogenetic variants are discussed.

## Results

Across all the 29 colorectal ASC samples, deriving mostly (22/29) from primary colorectal ASCs and their metastases (7/29), a total of 22/63 (34.9%) cancer-related genes were found to harbor SNVs, either missense, frameshift, stop gained (nonsense), or splice variants, and 11/44 (25.0%) cancer-related genes were found to harbor CNVs.

### Clinico-pathologic data

The median age at diagnosis was 73 years (range 42–92), and male to female ratio was 16/6. Regarding the stage at diagnosis, 5/22 (23%) were stage II, 8/22 were stage III (36%), and 7/22 (32%) were stage IV (information not available in 2/22 cases). Two patients with rectal disease received neoadjuvant therapy; the tumor regression grade of both lesions was scored TRG3 according to AJCC 2010.

The WHO 2019 grading system was applied to the glandular component of the 20 cases that were not subject to neoadjuvant therapy, 7/20 (35%) were low grade, and 13/20 (65%) were high grade. In 6/22 (27%) cases, the glandular component was ≥ 50% of the tumor.

Follow-up data were available for 17 patients; follow-up was 21 months (range 1 to 168 months). Twelve patients (71%) died of disease with a median survival of 10 months (range 1 to 72 months; five patients were stage IV, six patients were III, and one patient was stage II); 4 (24%) patients were alive at the last follow-up (one patient was stage III, and two patients were stage II); and one (5%) was alive with disease (stage IV).

### Molecular profiling of primary colorectal ASCs

**Fig. 1:**
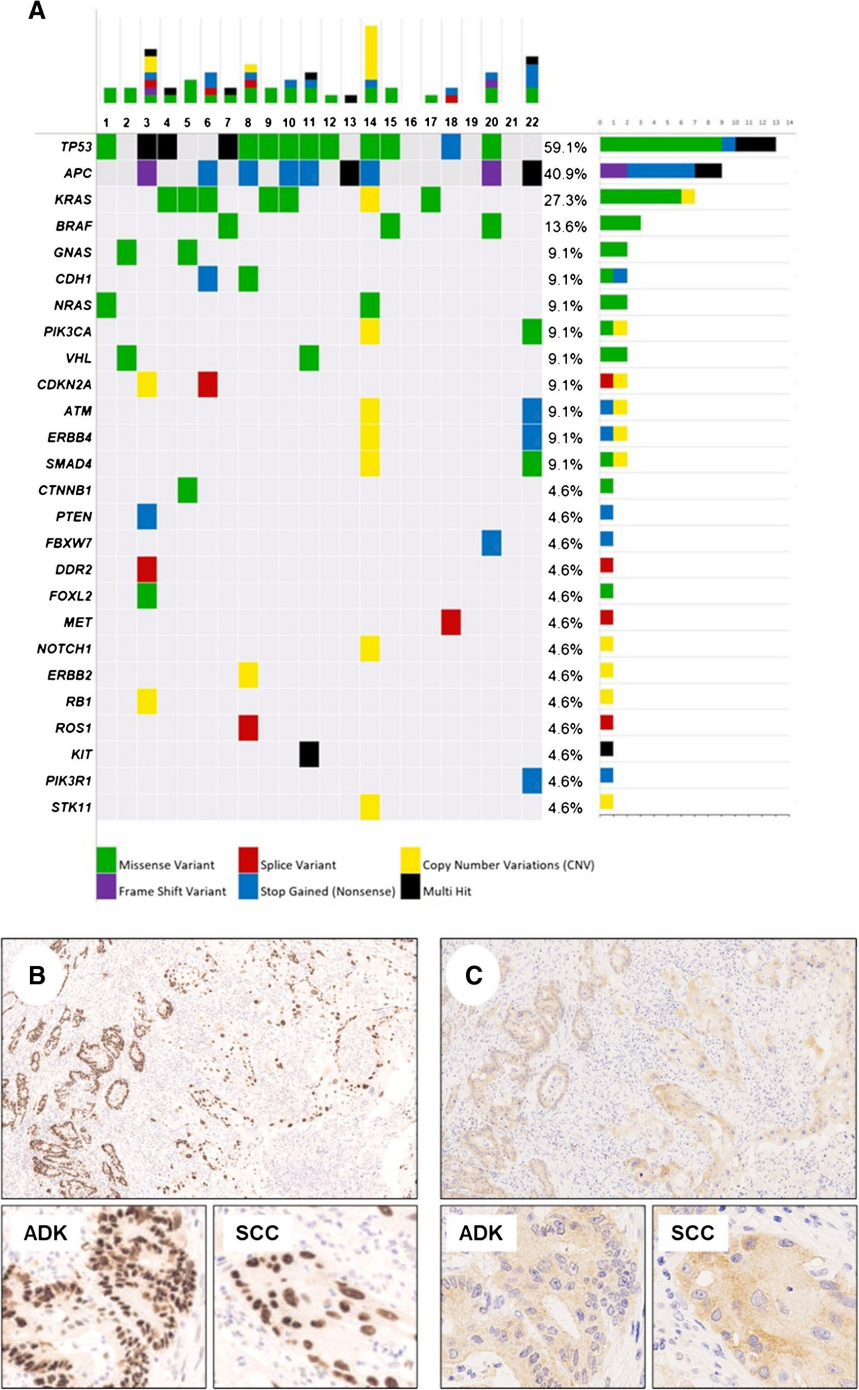
**A** Oncoplot summarizing the genomic findings (SNVs and CNVs) of the 22 analyzed primary colorectal ASCs. **B, C** Colorectal ASC showing a clonal mutator-phenotype p53 nulcear expression and ^V600E^*BRAF* cytoplasmic immunostaining in both components (ADK, adenocarcinoma; SCC, squamous cell carcinoma; case #15)

A comprehensive and summarizing representation of all detected alterations (single-nucleotide variants [SNVs] and copy number variations [CNVs]) of the 22 primary colorectal ASCs is provided in Fig. 1A.

In 20/22 (90.9%), primary ASCs at least one SNV were detected, and in 4/22 (18.2%), at least one CNV was detected.

The median number of genetic alterations (SNVs and/or CNVs) per sample was two and ranged from 0 to 9, with 8 of 22 (36.4%) samples harboring four or more alterations.

*TP53* SNVs were the most frequent genetic alterations observed in the series, occurring in 13/22 (59.1%) primary ASC samples. In three cases, the *TP53* gene harbored a double SNV. The most common variants were p.Arg248Trp and p.His179Leu, with four and three cases, respectively.

*APC* SNVs were observed in 9/22 (40.9%) of primary ASC samples. In two cases, the *APC* gene harbored a double SNV. The most common variants were p.Thr1556AsnfsTer3 (two cases) and p.Trp699Ter (two cases).

*KRAS* SNVs were observed in 6/22 (27.3%) of primary ASC samples. The most common variant was p.Gly12Asp (three cases).

*BRAF* SNVs were observed in 3/22 (13.6%) of primary ASC samples. The only variant detected was p.Val600Glu.

SNVs in other genes were detected at lower frequencies: *GNAS* (2/22, 9.1%), *CDH1* (2/22, 9.1%), *NRAS* (2/22, 9.1%), *VHL* (2/22, 9.1%), *PTEN* (1/22, 4.6%), *PIK3CA* (1/22, 4.6%), *CDKN2A* (1/22, 4.6%), *ATM* (1/22, 4.6%), *ERBB4* (1/22, 4.6%), *CTNNB1*(1/22, 4.6%), *FBXW7* (1/22, 4.6%), *SMAD4* (1/22, 4.6%), *DDR2* (1/22, 4.6%), *FOXL2* (1/22, 4.6%), *MET* (1/22, 4.6%), *ROS1* (1/22, 4.6%), *KIT* (1/22, 4.6%), and *PIK3R1* (1/22, 4.6%).

The following CNVs were observed: *ERBB2* loss (1/22, 4.6%), *KRAS* partial loss (1/22, 4.6%), *PIK3CA* loss (1/22, 4.6%), *CDKN2A* gain (1/22, 4.6%), *ATM* partial loss (1/22, 4.6%), *ERBB4* loss (1/22, 4.6%), *SMAD4* partial loss (1/22, 4.6%), *NOTCH1* gain (1/22, 4.6%), *RB1* loss (1/22, 4.6%), and *STK11* gain (1/22, 4.6%).

According to MMR status, 21/22 (95.5%) were MMRp, and 1/22 (4.5%) (case #7) showed loss of MLH1 and PMS2 by IHC (MMRd). This latter case was also characterized by a *BRAF* p.Val600Glu mutation.

p53 expression was aberrant in 12/13 (92%) of the *TP53*-mutated cases, with a strong and diffuse staining pattern in 11 cases and a complete loss of expression in one case. No difference in p53 expression was observed between the glandular and squamous components (Fig. 1B).

^V600E^*BRAF* IHC was positive in the three *BRAF*-mutated cases, and no difference was observed between the two components (Fig. 1C).

### Molecular profiling of metastases of colorectal ASCs

The seven metastatic lesions analyzed were matched with four (cases #5, #9, #11, and #20) of the primary ASCs and were distributed as follows: one metachronous skin metastasis and one metachronous liver metastasis matched with primary #5; one synchronous lymph node metastasis and two metachronous metastases (cutaneous and omental) matched with primary #9; one synchronous omental metastasis matched with primary #11; and one synchronous lymph node metastasis matched with primary #20. All the 7 metastatic lesions were MMRp.

Table 1 summarizes the genetic alterations found in the metastatic lesions and matched primary ASCs. Limited differences in SNVs were observed between matched primary and metastatic tumors.

**Table 1:** Genetic alterations in metastatic and matched primary ASCs of our series

	Primary	Metastasis n.1	Metastasis n.2	Metastasis n.3
#5	SNVs: *KRAS, CTNNB1* and* GNAS*	Skin (M) SNVs:* KRAS, CTNNB1* and* GNAS*	Liver (M) SNVs:* KRAS, CTNNB1* and* GNAS*	*-*
#9	SNVs: *TP53* and* KRAS*	Lymph node (S) *SNVs: TP53* and* KRAS*	Skin (M) SNVs: *TP53* and *KRAS*	Peritoneum (M) SNVs:* TP53, KRAS,* and* SMAD4*
#11	SNVs*: TP53, APC, VHL,* and* KIT*	Peritoneum (S) SNVs:* TP53, APC, *and *PTEN*	*-*	*-*
#20	SNVs:* TP53, APC, BRAF, *and *FBXW7*	Lymph node (S) SNVs:* TP53, APC, BRAF,* and *FBXW7* CNVs:* MYC gain*	*-*	*-*

*S*, synchronous; *M*, metachronous; *SNV*, single-nucleotide polymorphism; *CNV*, copy number variation

## Discussion

This study gathers insight into the molecular landscape of colorectal ASC, which is a rare subtype of colorectal adenocarcinoma. Due to the rarity of this subtype, molecular alteration data in colorectal ASC are scarce. We are the first to provide a mutational analysis of colorectal ASC using an extended set of cancer-related genes including those which are frequently mutated in conventional CRC.

In our series, *TP53* (59.1%) is the most commonly mutated gene in colorectal ASC, followed by *APC* (40.9%) and *KRAS* (27.3%). Several studies provide genomic data on conventional CRC and identify these three genes as the most frequently mutated in this cancer type. However, the reported mutation rates of *TP53, APC*, and *KRAS* are variable, possibly due to the different platforms and assays used in previous studies and to the different ethnicities of the populations. According to TCGA data [8], *APC* mutation (71%), followed by *TP53* (54%) and *KRAS* (42%) mutations, is the most frequent in conventional CRC. By using targeted NGS, Lee and colleagues [9] showed that *TP53* (67%) is the most commonly mutated gene in CRC, followed by *APC* (60%) and *KRAS* (47%). In a recent Chinese study [10], 32 CRC were investigated by whole exome sequencing (WES), revealing a mutation rate of 59.38% in *APC* and of 50% in *TP53*. Because *KRAS* mutations are predictors of resistance to anti-EGFR antibodies [11], their prevalence in CRC has been extensively investigated, and it is estimated to be approximately 35–45%, similarly to our results. However, the prognostic value of *KRAS* remains controversial. While some studies showed no prognostic role of *KRAS* mutations, others demonstrated an association with shorter disease-free survival (DSF) and overall survival (OS) and with liver metastases [12]. The rate of *NRAS* mutations*,* which are also well-established drivers of resistance to anti-EGFR therapy, was comparable between ASCs and conventional CRCs.

^V600E^*BRAF* mutation is an established negative prognostic marker and has also relevant therapeutic implications [13]. In our series, the prevalence of ^V600E^*BRAF* mutations is higher than in conventional CRC (13.7% vs 10–5%), possibly due to the enrichment of metastatic cases in our case series. Among CRCs, the rate of *BRAF* mutations is significantly higher in the metastatic setting (stage IV) rather than in stage II–III (15–20% vs 10–5%). Of note, no ^non−V600E^*BRAF* mutations were detected.

Activating *GNAS* mutations are common in mucinous neoplasms such as intraductal papillary mucinous neoplasms (IPMNs) and low-grade appendiceal mucinous neoplasms (LAMNs), and several studies have shown that *GNAS* mutant CRCs often contain a mucinous component [14]. With the limitation posed by the small sample size, our data suggest that the *GNAS* mutation rate is higher in colorectal ASC than in conventional CRC (former studies reported values ranging from 0 to 3.1% in CRC). Additionally, a previous report has showed a *GNAS* mutation in a Lynch syndrome-associated ASC [5]. In our series, *GNAS* mutations were detected in 9.1% of colorectal ASCs. Two SNVs involving codon 201 of the *GNAS* gene were identified in our series (i.e., p.Arg201His and p.Arg201Cys*)*, which have been described in previous works as the two most common *GNAS* activating mutations[14].

With regard to CNVs, *ERBB4* loss and *ERBB2* loss are extremely rare in conventional CRC. *ERBB4* loss is most frequently found in invasive breast carcinoma and prostate adenocarcinoma, while *ERBB2* loss is found in ovarian high-grade serous carcinoma and invasive breast carcinoma. RB loss is an infrequent event in CRC; however, it is a common molecular event in colorectal neuroendocrine carcinoma. *NOTCH1* amplifications are present in 0.08% of all colon carcinoma patients; *NOTCH1* alterations are thought to drive progression and metastatic seeding by TGF-beta signaling[15].

Microsatellite instability (MSI) is present in approximately 15% of CRCs and can be caused by germline and/or somatic mutations or epigenetic silencing (MLH1 gene promoter hypermethylation) [6]. In our case series, only one case showed an MMRd/MSI profile, together with a ^V600E^*BRAF* mutation, indicating the sporadic nature of the neoplasm.

The molecular profiles of the matched primary tumors and metastases were similar but in one case, possibly due to the selective pressure on a neoplastic clone. Intratumoral molecular heterogeneity fuels resistance to targeted and immune therapies.

Despite being limited by the small sample size, due to the rarity of this histotype, our study has shown that the genomic profile of colorectal ASC is similar to that of conventional CRC, with an overlapping prevalence of driver mutations, such as *TP53*, *APC*, *KRAS*, and *BRAF*. Notably, an enrichment of *GNAS* mutations, a lower prevalence of *PIK3CA* mutations, and the presence of infrequent CNVs in comparison to CRC were observed. Further studies are warranted on the molecular landscape accounting for the aggressive behavior of colorectal ASC, as well as to identify potential therapeutic targets.

## Data Availability

The data that support the findings of this study are available from the corresponding authors (AV, MF), upon reasonable request.
